# Calcium/Calmodulin-Mediated Defense Signaling: What Is Looming on the Horizon for AtSR1/CAMTA3-Mediated Signaling in Plant Immunity

**DOI:** 10.3389/fpls.2021.795353

**Published:** 2022-01-11

**Authors:** Peiguo Yuan, Kiwamu Tanaka, B. W. Poovaiah

**Affiliations:** ^1^Department of Horticulture, Washington State University, Pullman, WA, United States; ^2^Department of Plant Pathology, Washington State University, Pullman, WA, United States

**Keywords:** AtSR1/CAMTA3, Ca^2+^ signaling, CaMs/CMLs, CBL-CIPK, CPKs, MAPKs, plant immune response

## Abstract

Calcium (Ca^2+^) signaling in plant cells is an essential and early event during plant-microbe interactions. The recognition of microbe-derived molecules activates Ca^2+^ channels or Ca^2+^ pumps that trigger a transient increase in Ca^2+^ in the cytoplasm. The Ca^2+^ binding proteins (such as CBL, CPK, CaM, and CML), known as Ca^2+^ sensors, relay the Ca^2+^ signal into down-stream signaling events, e.g., activating transcription factors in the nucleus. For example, CaM and CML decode the Ca^2+^ signals to the CaM/CML-binding protein, especially CaM-binding transcription factors (AtSRs/CAMTAs), to induce the expressions of immune-related genes. In this review, we discuss the recent breakthroughs in down-stream Ca^2+^ signaling as a dynamic process, subjected to continuous variation and gradual change. AtSR1/CAMTA3 is a CaM-mediated transcription factor that represses plant immunity in non-stressful environments. Stress-triggered Ca^2+^ spikes impact the Ca^2+^-CaM-AtSR1 complex to control plant immune response. We also discuss other regulatory mechanisms in which Ca^2+^ signaling activates CPKs and MAPKs cascades followed by regulating the function of AtSR1 by changing its stability, phosphorylation status, and subcellular localization during plant defense.

## Introduction—Calcium Signaling Cascades Control Plant Defense Responses

**FIGURE 1:**
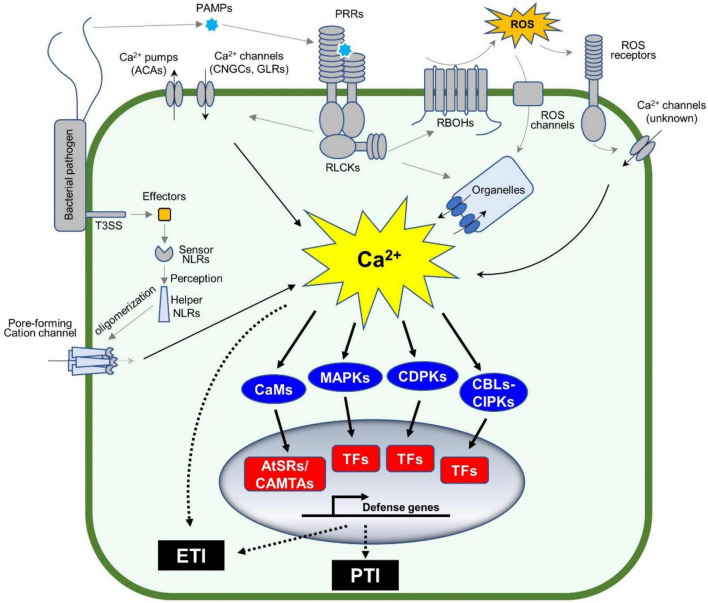
Calcium signaling cascades control plant defense gene expression. Plants sense pathogens (e.g., bacteria in the figure) through pattern recognition receptors (PRRs) by recognizing the pathogen-associated molecular patterns (PAMPs). The activated PRRs phosphorylate themselves and other proteins including receptor-like cytoplasmic kinases (RLCKs). The phosphorylated RLCKs activate Ca^2+^ channels or Ca^2+^ pumps, leading to the dynamic changes in cytosolic Ca^2+^ concentration, while the activated RLCKs phosphorylate RBOHs to produce apoplastic ROS accumulation, which indirectly induces the Ca^2+^ influx in the plant cell. Ca^2+^ sensors, such as CaM/CML, CBLs, and CDPKs, together with MAPKs, decode the pattern-triggered Ca^2+^ spiking into downstream signaling. CBL-activated CIPKs and CDPKs phosphorylate a variety of transcription factors (TFs), such as WRKYs, to induce the expression of defense-related genes. In addition, the TFs (e.g., AtSRs/CAMTAs) activated by Ca^2+^-bound CaMs are involved in Ca^2+^-mediated immune response. The entire process is important to activate pattern-triggered immunity (PTI). On the other hand, pathogens secrete proteins, so-called effectors, into the plant cell to repress the plant immune system, also known as ETS. However, the effectors are recognized by intracellular nucleotide-binding domain and leucine-rich repeat proteins (NLRs) to activate a strong and rapid immune response, known as effector-triggered immunity (ETI). The activated NLRs oligomerize and form a non-canonical Ca^2+^-permeable channel in the plasma membrane, to induce a strong Ca^2+^ influx (Ca^2+^ burst) followed by PCD.

Plant immune systems rely on multiple layers of recognition systems to confer full protection to pathogen attack. For example, pattern recognition receptors (PRRs) on cell surfaces recognize pathogen-associated molecular patterns (PAMPs) or damage-associated molecular patterns (DAMPs), each of which is derived from pathogenic microbes or damaged plants themselves (Jones and Dangl, 2006; Tanaka and Heil, 2021). This leads to PRR-mediated immunity, or so-called pattern-triggered immunity or PTI (Denancé et al., 2013; Yuan et al., 2017). Pathogens secrete virulence determinants referred to as effectors to inhibit PTI or other plant physiological responses. However, some effectors are recognized by intracellular nucleotide-binding domains and leucine-rich repeat proteins (NLRs), which result in NLR-mediated immunity, or so-called effector-triggered immunity or ETI (Jones et al., 2016). Notably, cellular responses during both PTI and ETI involve dynamic changes in cytosolic Ca^2+^ concentrations (Zhivotovsky and Orrenius, 2011; Yuan et al., 2020, 2021). Changes in cytosolic Ca^2+^ concentrations are sensed by the Ca^2+^-signaling toolkit (Marcec et al., 2019) e.g., Ca^2+^ sensors and/or decoders [calmodulin (CaM), CaM-like proteins (CML), “calcineurin B-like protein” (CBL)-“CBL-interacting protein kinases” (CBL-CIPK), and calcium-dependent protein kinases (CPKs or CDPKs)], which, together with mitogen-activated protein kinases (MAPKs) activation, coordinate the transcriptional reprogramming of defense genes through activation of various transcript factors (TFs) (Figure 1).

The dynamic changes in cytosolic Ca^2+^ concentrations are an early event during immune responses, where the Ca^2+^ channels and Ca^2+^ pumps are activated to form specific Ca^2+^ signatures to each stimulus (Marcec et al., 2019; Marcec and Tanaka, 2022). For example, cyclic nucleotide-gated ion channel 2 (CNGC2) forms a heteromeric cation channel with CNGC4 playing an important role in the flg22-induced rise of Ca^2+^ in plant cells. In this event, PRR-activated receptor-like cytoplasmic kinases (RLCKs), e.g., botrytis-induced kinase 1 (BIK1), activate the Ca^2+^ channel through the phosphorylation of CNGC4, but not CNGC2. In addition, BIK1 also phosphorylates CaM7 that, in turn, binds to the IQ motif in CNGC2 and CNGC4 to suppress the activity of the heteromeric Ca^2+^ channel (Tian et al., 2019), which could be a desensitization mechanism to regulate the immune response. The application of H_2_O_2_ induces Ca^2+^ influx, where the hydrogen peroxide-induced Ca^2+^ increase 1 (HPCA1), which functions as an extracellular H_2_O_2_ receptor and is required for H_2_O_2_-induced Ca^2+^ rise (Figure 1). Recently, the NLR receptor hopz-activated resistance 1 (ZAR1) resistome was revealed to form a Ca^2+^-permeable channel to trigger the programmed cell death (PCD) (Wang et al., 2019; Bi et al., 2021). Another study revealed that the active NLR, N requirement gene 1 (NRG1), also forms puncta in the plasma membrane, which is a non-selective cation channel leading to permeability for Mg^2+^ and Ca^2+^, but not Cl^–^ (Jacob et al., 2021). These channels based on ZAR1 or NRG1 likely cause a strong, prolonged Ca^2+^ signature as a Ca^2+^ burst, which plays a central role during ETI-mediated PCD (Jacob et al., 2021). Most studies on Ca^2+^ channels focus on the plasma membrane-localized channels, since Ca^2+^ channel blocker, La^3+^ or Gd^3+^, can suppress the NLRs-forming Ca^2+^ channels. It is not clear how the organelle membrane localized Ca^2+^ channels are involved in plant immune response. Currently, there are many unanswered questions, for example, how Ca^2+^ burst induces PCD and other ETI. Ca^2+^/CaM-binding transcription factors (CAMTAs) or *Arabidopsis thaliana* signal responsives (AtSRs) could be a sensor of the Ca^2+^ burst (Yuan et al., 2021) as described in the Section 2 below.

AtSR1/CAMTA3 is a transcriptional regulator in response to biotic stress-induced Ca^2+^ changes, and plays a suppressor role in the plant immune system (Du et al., 2009) since *atsr1/camta3* mutants show an autoimmune phenotype, including elevated salicylic acid (SA) and reactive oxygen species (ROS) concentrations, and enhanced resistance to bacterial and fungal pathogens (Galon et al., 2008; Du et al., 2009). AtSR1/CAMTA3 requires CaM binding for its activation, suggesting a role for Ca^2+^ in repressing its function in plant immunity. However, accumulating evidence suggests that AtSR1/CAMTA3 functions as more than a negative regulator in the plant immune system. In this review, we summarize the recent progress related to studies on AtSRs/CAMTAs during plant defense responses that could help in our understanding of their unique roles in the plant immune system.

## AtSR1/CAMTA3 Is a Central Signaling Component in Plant Immune Responses

### CaM-Mediated Regulation of AtSR1/CAMTA3

**FIGURE 2:**
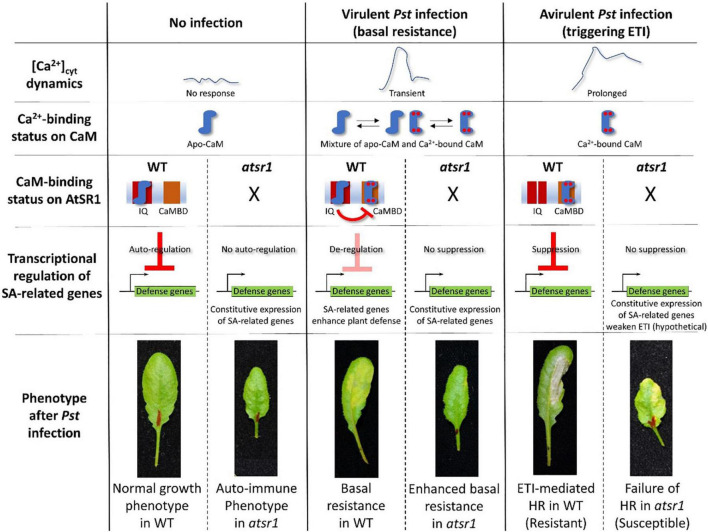
AtSR1/CAMTA3 acts as a signaling switch with dependence on Ca^2+^-binding status of CaM during plant immune response. In non-stressful environments, there is no [Ca^2+^]_*cyt*_ dynamic. Hence, the status of CaM is not in a saturated phase at four Ca^2+^ binding sites, designated as apo-CaM. Apo-CaM interacts with the IQ motifs of AtSR1. This CaM-AtSR1 complex may have an effect on the stability of AtSR1, which suppresses the transcriptional expression of defense-related genes (e.g., EDS1, ICS1, and *NPR1*) as an autoregulator. Thus, suppression of plant immunity maintains normal plant growth and development. Under the virulent pathogen infection, [Ca^2+^]_*cyt*_ is transiently increased, which confers a status of mixture of Apo-CaM and Ca^2+^-saturated CaM. Ca^2+^-saturated CaM interacts with the CaMBD of AtSR1, while Apo-CaM interacts with the IQ motifs. This CaM-AtSR1 complex loses its binding activity to the *cis*-regulatory element “CGCG,” and de-regulates the transcriptional expression of the defense-related genes. This eventually activates the basal resistance or PTI. Under avirulent pathogen infection, [Ca^2+^]_*cyt*_ is constitutively increased, which leads to stable binding of Ca^2+^ ions to CaM. The Ca^2+^-saturated CaM interacts only with the CaMBD of AtSR1. This CaM-AtSR1 complex regulates the ETI-triggered PCD. In the *atsr1* mutant, due to the loss of negative regulation of the *NPR1* transcription by AtSR1, the plants constitutively activate basal immune response, resulting in the autoimmune phenotype. Under pathogen infection in the *atsr1* mutant due to the lack of fine-tuning for transcriptional regulation of defense-related genes, the immune response is disturbed in the mutants, i.e., enhanced basal resistance and failure of ETI-induced PCD to the virulent and avirulent bacterial pathogens, respectively. Refer to Yuan et al. (2021) for further details.

AtSR1/CAMTA3 is known as a Ca^2+^/CaM-regulated transcription factor involved in transcriptional reprogramming during plant immune response. AtSR1 binds to the CGCG *cis*-regulatory element in the promoter of *enhanced disease susceptibility 1 (EDS1), non-race-specific disease resistance1 (NDR1)*, and *non-expresser of PR genes1 (NPR1)* to modulate their expression in SA-mediated plant immunity (Yuan et al., 2018b,^2021^). AtSR1 also regulates *isochorismate synthase 1 (ICS1)* to suppress plant immunity at both 20*^o^*C and 28*^o^*C (Du et al., 2009). AtSR1 contributes to systemic acquired resistance (SAR) through the regulation of NDR1 expression (Nie et al., 2012). A recent study revealed that AtSR1 is required to establish a proper plant immune response to basal resistance or ETI-triggered PCD, also known as hypersensitive response (HR) cell death (Yuan et al., 2021; Figure 2).

The AtSRs family contains different types of CaM-binding domains (CaMBDs). CaM binding to AtSR1 is essential for the function of AtSR1 (Reddy et al., 2011). Yuan et al. (2021) demonstrated that a loss-of-function mutation on CaMBD did not complement the *atsr1* mutant phenotype, i.e., failed to suppress the *EDS1* expression, suggesting that CaMBD is essential for the AtSR1 function. In contrast, a gain-of-function mutant, *atsr1-4D*, in which a mutation was located at the first IQ motif, displayed constitutive down-regulation of transcriptional expressions of *EDS1* and *NDR1* (Nie et al., 2012). Given that the first IQ motif binds to apo-CaM (Ca^2+^ free CaM), and the CaMBD binds to Ca^2+^-bound CaM (Yuan et al., 2021), AtSR1 acts as a signaling switch with dependence on the level of cytosolic Ca^2+^ concentration. The complementation with double mutation at the IQ motif and CaMBD restored the plant phenotype similar to WT, which indicated that the IQ motif and the CaMBD in AtSR1 interact during the plant immune response (Kim et al., 2017; Yuan et al., 2018c; Figure 2).

Recently, AtSRs were also reported to negatively regulate the pipecolic acid (Pip)-mediated plant immune response (Kim et al., 2020; Sun et al., 2020). The biosynthesis of Pip was activated in *camta1/2/3* and the transcriptional expressions of *agd2-like defense response protein 1 (ALD1)* and *flavin-dependent monooxygenase 1 (FMO1)*, which both encode two pip biosynthesis enzymes, were greatly induced in *camta1/2/3*. Moreover, CBP60g and SARD1 were identified to regulate Pip production by ALD1 and FMO1; also, AtSR1 protein interacted with the CGCG box in the promoter of *calmodulin binding protein 60g (CBP60g)*, but not that of *SAR deficient 1 (SARD1)*, to repress the CBP60g expression (Sun et al., 2020). In addition, the *atsr1* auto-immunity phenotype was compromised by not only *sard1 cbp60g* double mutant, but *ald1 fmo1* double mutant as well (Sun et al., 2020). These observations suggest that SA and *N-*Hydroxypipecolic acid (NHP) crosstalk to mediate plant immune response. This idea is supported by the observation that the application of Pip to plant leaf promoted NPR1 stability (Kim et al., 2020). How AtSR1 with or without Ca^2+^-bound CaM regulates pip biosynthesis remains to be determined.

**FIGURE 3:**
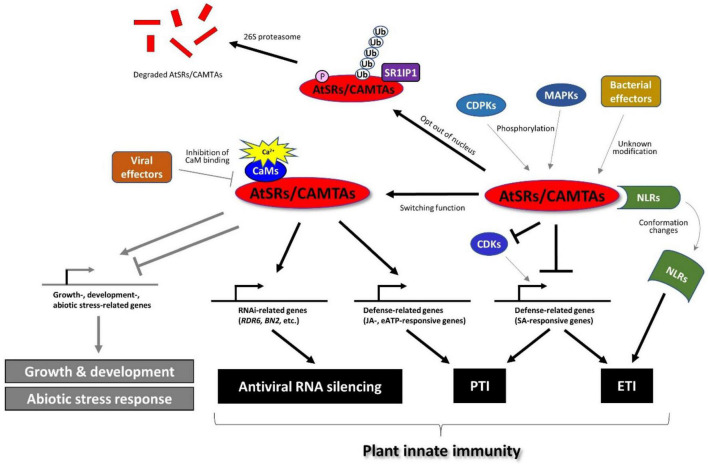
AtSRs/CAMTAs play the role of a hub in plant immune response. AtSRs are the suppressor of plant immunity and repress defense-related gene expressions through the interaction with the “CGCG” box in their promoter. Pathogen infection induces Ca^2+^ influx as described in Figure 1, which impacts the Ca^2+^-CaM-AtSRs complex and subsequently removes the suppressor of AtSRs in SA-based plant immunity to induce the defense-related gene expression (e.g., EDS1, and NPR1, etc.). However, effector proteins derived from the bacterial pathogens (e.g., *Pst* carrying AvrRpt2 or AvrRps4) modify AtSRs, which is sensed by NLRs, e.g., DSC1 and DSC2, followed by inducing ETI-based plant defense response. In addition, the rise of cytoplasmic Ca^2+^ activates MPKs and CPKs, which phosphorylate AtSRs, resulting in the export of AtSRs from the nucleus and eventually the degradation of AtSRs. In contrast, AtSRs act as transcriptional activators for plant immune response by reprograming the transcription of JA- and eATP-responsive genes. In addition, CaM-AtSRs interaction is targeted by viral effectors, e.g., V2 derived from the geminivirus CLCuMuV, which regulates RNAi-mediated immune response against viral infection. In other respects, AtSRs also play an important role in plant growth, development, and abiotic stress responses, which are not discussed in this review.

A recent study revealed that AtSR1/CAMTA3 is more than a transcriptional repressor in the plant immune response (Figure 3). For example, AtSR1 was found to mediate DAMP-induced signaling, whereas AtPep1-induced reprograming of JA-responsive genes requires functional AtSR1 (Yuan et al., 2020). Extracellular ATP (eATP) induces defense-related transcriptomes in which the CGCG *cis*-regulatory element was highly enriched in the eATP-responsive promoters (Jewell and Tanaka, 2019; Jewell et al., 2019). Interestingly, AtSR1/CAMTA3 is required for defense gene induction in response to eATP treatment. A recent report demonstrated that CaM-AtSR1 interaction regulates RNAi-mediated immune response against viral infection, where CaM3- and CaM6-bound AtSR1 positively regulate the RNAi system (Wang Y. et al., 2021). AtSR1 interacts with the promoter of *RNA-dependent RNA polymerase 6* (*RDR6*) and *bifunctional nuclease-2* (*BN2*) to induce the transcriptional expression of RDR6 and BN2, respectively. RDR6 is known to convert single-stranded (ss) RNA into double-stranded (ds) RNA to induce RNA silencing (Harmoko et al., 2013), while BN2 is a ribonuclease that degrades microRNAs to activate RNAi. Given that the virus enters into plant cells through natural wound sites and herbivory of insects, wound-induced Ca^2+^ influx in plant cells may promote the interaction between CaMs and AtSR1 to activate the antiviral RNAi system. In contrast, the virus (a geminivirus CLCuMuV in the reported case) employs an effector protein, V2, to impair the interaction between CaM3 and AtSR1 to suppress plant defense (Wang Y. et al., 2021), which provides corroborative evidence of AtSR1-mediated immune response against the virus.

### Phosphorylation-Based Regulation of AtSR1/CAMTA3

Facing pathogen challenge, it is necessary for the activation and establishment of plant immune response to overcome the suppressor of AtSR1 (Yuan et al., 2018b). The pathogen infection promotes the degradation of AtSR1 through proteasome-mediated pathway where AtSR1 interaction protein 1 (SR1IP1) is a substrate-adaptor for cullin3-based E3 ubiquitin ligase that likely regulates the AtSR1 degradation (Zhang et al., 2014). A recent study revealed that MAPKs cascade contribute to proteasome-mediated turnover of AtSR1 (Jiang et al., 2020). The application of flg22-induced phosphorylation of AtSR1 (mediated by mitogen-activated protein kinases 3 (MPK3) and MPK6, but not MPK4) further results in destabilization of the AtSR1 protein. Recently, the CPKs or CDPKs are also revealed to be involved in pathogen-induced phosphorylation events of AtSR1 (Jiang et al., 2020). The *Arabidopsis* genomic DNA encodes a large number of the CPK gene family, which contains 34 members and is grouped into four subgroups. The stability of the AtSR1 protein was greatly disrupted in the co-expression of CPK1, CPK2, and CPK5, where only CPK5 was reported to be activated by flg22 (Jiang et al., 2020). Taken together, MAPKs and CDPKs appear to be essential for the phosphorylation and degradation of AtSR1 during plant-microbe interactions, although AtSR1 is not phosphorylated directly by CPK5 *in vitro* (Jiang et al., 2020).

Controlling the subcellular localization is another way to sequester the negative function of AtSR1 in plant immunity. AtSR1 contains two nuclear localization sequences (NLSs) (Yang and Poovaiah, 2002). The flg22 triggers the subcellular re-localization of the AtSR1 from the nucleus to the cytoplasm, where MAPKs regulate AtSR1 phosphorylation to export AtSR1 out of the nucleus (Jiang et al., 2020), although the MAPK-independent regulation remains to be studied. In addition, the protein phosphatase, such as protein phosphatase 2C (PP2C), is a major regulator of plant immune response. However, the role of phosphatase in AtSR1-mediated plant defense remains to be studied.

### AtSR1/CAMTA3 as a Guardee

RIN4 is a well-studied guarded effector target, or so-called “guardee.” However, there are more potential guardees. For example, EXO70B1, a subunit of the exocyst complex, can be a guardee that can activate ETI *via* the truncated NLR TN2, where CPK5 is required for this TN-mediated immunity (Liu et al., 2017). Also, AtSR1/CAMTA3 was reported as a guardee based on accumulating evidence (Liu et al., 2017). Two toll/interleukin-1 receptor (TIR)-NLR proteins, called dominant suppressor of *camta3* number 1 (DSC1) and DSC2 directly interacts with AtSR1, where DSC1 and DSC2 guard the guardee AtSR1. Upon infection of avirulent *Pst* strains carrying avrRps4 and avrRpt2, a rapid degradation of AtSR1 is induced probably following phosphorylation and/or ubiquitination of AtSR1. The degradation of AtSR1 contributes to the activation of downstream immune responses by de-repression of defense-related genes, e.g., *EDS1* and *NDR1* (Figure 3). This notion explains how the auto-immunity phenotype in *atsr1* requires DSC1 and DSC2 (Lolle et al., 2017). AtSR1 degradation leads to the activation of NLRs-mediated plant immunity. Thus, it is reasonable to hypothesize that a modification of AtSR1 by an unidentified pathogen effector, such as phosphorylation and proteolysis, is sensed by the DSC1 and DSC2, or other unknown NLRs. It would be interesting to know if DSC1 and/or DSC2 might sense the phosphorylated AtSR1, by MPK3, MPK6 and CPK5, as mentioned above. In other respects, DSC1 and DSC2 were identified to be localized in the nucleus; hence, they may also sense the subcellular re-localization of the AtSR1.

### Mediator-Associated Regulation of AtSR1/CAMTA3

In eukaryotic cells, RNA polymerase II (Pol II) is required for most transcriptions of general protein-coding genes and several non-coding RNA (ncRNAs) genes (Palazzo and Lee, 2015). The mediator is the highly conserved, large multi-subunit regulator, which works together with RNA Pol II, TFs, and co-TFs to activate the gene expression upon perception of environmental and developmental stimuli. The genetic analysis revealed that cyclin-dependent kinase 8 (CDK8), a mediator subunit, modulates the AtSR1-regulated SA signaling pathway in plant immune response (Huang et al., 2019), where a strong autoimmune phenotype of the triple mutant *camta1/2/3* (*atsr2/4/1*) is partially suppressed in the *cdk8* null mutant. Further study revealed that CDK8 regulates SA biosynthesis genes, such as *ICS1* and *EDS5*, probably *via* AtSRs-mediated transcriptional reprogramming, although a detailed mechanism of how CDK8 regulates AtSRs remains to be studied (Huang et al., 2019). Another report revealed that CDK8 recruits NPR1 and WRKY18 to promote defense gene expression, where SA further facilitates the interaction between CDK8 and NPR1. In addition, CDK8 interacts with other TFs, TGA5 and TGA7, together with NPR1, to induce PR1 expression (Chen et al., 2019). It is interesting to speculate that AtSR1 is the key regulator of CDKs during plant immune responses, but the mechanisms remain to be investigated further.

### AtSR1 as Positive Regulator of Plant Growth and Development

Facing pathogen infection, a plant reduces the resource and energy for growth and development, and switches to activate and establish plant immune response to restrict the invading pathogen (Denancé et al., 2013). During the resting stage, AtSR1 represses the plant defense to maintain plant growth and development (Yuan et al., 2018a). Hence, AtSR1 is involved in promoting growth. AtSR1 was found to be involve in IAA and BR signal transduction. AtSR1 interacts with the “CGCG” box in the promoter of *IAA1 and IAA19* to regulate their expressions, and regulates *DWF4* expression through binding to its promoter (Yuan et al., 2018a). However, the mechanisms involved are not clearly understood.

## Conclusion and Future Perspectives

In recent years, a great deal of progress has been made in understanding Ca^2+^ channels and the associated down-stream signaling. However, there are still several key questions which remain to be addressed. Although many Ca^2+^ sensors have been identified that regulate plant immune response, the role of CMLs is still unclear and remains to be studied. In addition, the pathogen-triggered modifications (such as phosphorylation and ubiquitination) of AtSR1 suppresses its negative function in plant immunity, however, the molecular mechanism of recovery of AtSR1 after successful prevention of pathogen infection needs to be addressed. There are several major questions: (1) The role of AtSR1 in plant growth remains unclear (Yuan et al., 2018a). (2) Further studies need to be carried out on AtSR1-mediated plant defense against herbivory. (3) The molecular mechanism of AtSR1 decoding Ca^2+^ signaling through IQ motif and/or CaMBD still needs to be addressed. (4) How AtSR1 crosstalks with hormonal pathways, such as gibberellins (GAs), brassinosteroids (BRs), ethylene (ET) and auxin is not understood.

Since the focus of this special issue is on signaling in plant biotic interactions, it is appropriate to point out that Ca^2+^/CaM-mediated signaling plays a unique role in both pathogenic (e.g., AtSR1 discussed above) and symbiotic interactions [Ca^2 +^/CaM-dependent protein kinase (CCaMK)] in plants. It is well recognized that CCaMK, a Ca^2+^/CaM-binding protein (Patil et al., 1995), plays a key role in fungal and bacterial symbioses (Patil et al., 1995; Gleason et al., 2006; Routray et al., 2013; Wang T. et al., 2021). However, the focus of this review is on plant immune response, hence any discussion on symbioses is beyond the scope of this review.

## Author Contributions

PY, KT, and BP were involved in writing this review. All authors contributed to the article and approved the submitted version.

## Conflict of Interest

The authors declare that the research was conducted in the absence of any commercial or financial relationships that could be construed as a potential conflict of interest.

## Publisher’s Note

All claims expressed in this article are solely those of the authors and do not necessarily represent those of their affiliated organizations, or those of the publisher, the editors and the reviewers. Any product that may be evaluated in this article, or claim that may be made by its manufacturer, is not guaranteed or endorsed by the publisher.
